# Effects of treatment changes on asthma phenotype prevalence and airway neutrophil function

**DOI:** 10.1186/s12890-017-0511-6

**Published:** 2017-12-04

**Authors:** Collin R. Brooks, Christine J. Van Dalen, Elizabeth Harding, Ian F. Hermans, Jeroen Douwes

**Affiliations:** 1 Centre for Public Health Research, Massey University Wellington Campus, Private Box 756, Wellington, 6140 New Zealand; 2 Malaghan Institute of Medical Research, Wellington, New Zealand

**Keywords:** Asthma, Inflammatory phenotype, Induced sputum, Neutrophils, Eosinophils

## Abstract

**Background:**

Asthma inflammatory phenotypes are often defined by relative cell counts of airway eosinophils/neutrophils. However, the importance of neutrophilia remains unclear, as does the effect of ICS treatment on asthma phenotypes and airway neutrophil function. The purpose of this study was to assess asthma phenotype prevalence/characteristics in a community setting, and, in a nested preliminary study, determine how treatment changes affect phenotype stability and inflammation, with particular focus on airway neutrophils.

**Methods:**

Fifty adult asthmatics and 39 non-asthmatics were assessed using questionnaires, skin prick tests, spirometry, exhaled nitric oxide (FENO) measurement, and sputum induction. Twenty-one asthmatics underwent further assessment following treatment optimisation (*n* = 11) or sub-optimisation (*n* = 10).

**Results:**

Forty percent (20/50) had eosinophilic asthma (EA) and 8% had neutrophilic asthma. EA was associated with increased FENO, bronchodilator reversibility (BDR) and reduced lung function (*p* < 0.05). Following optimisation/sub-optimisation, the EA/NEA (non-eosinophilic asthma) phenotype changed in 11/21 (52%) asthmatics. In particular, fewer subjects had EA post treatment optimisation, but this was not statistically significant. However, a significant (*p* < 0.05) reduction in FENO, ACQ7 score, and BDR was observed after treatment optimisation, as well as an increase in FEV_1_-% predicted (*p* < 0.05). It was also associated with reduced eosinophils (p < 0.05) and enhanced neutrophil phagocytosis (*p* < 0.05) in EA only, and enhanced neutrophil oxidative burst in both EA and NEA (*p* < 0.05).

**Conclusions:**

In this community based population, non-eosinophilic asthma was common, less severe than EA, and at baseline most asthmatics showed no evidence of inflammation. In the nested change in treatment study, treatment optimisation was associated with reduced sputum eosinophils, improved symptoms and lung function, and enhanced neutrophil function, but a significant reduction in EA could not be demonstrated.

**Trial registration:**

The nested change in treatment component of this study is registered at the Australia and New Zealand Clinical Trial Registry (www.ANZCTR.org.au) ACTRN12617001356358. Registration date 27/09/2017. Retrospectively registered.

**Electronic supplementary material:**

The online version of this article (10.1186/s12890-017-0511-6) contains supplementary material, which is available to authorized users.

## Background

Airway inflammation is a defining feature of asthma, often associated with eosinophilic, TH2-mediated immunopathology [1]. However, asthma may occur in the absence of airway eosinophils in approximately 50% of cases [2]. Non-eosinophilic asthma (NEA) can be observed across the spectrum of severity, is associated with distinct pathological features [3] and appears less responsive to inhaled corticosteroids (ICS) [4]. Identification and further characterisation of inflammatory asthma phenotypes may therefore help guide asthma treatment and contribute to novel treatment options for corticosteroid resistant asthma.

Non-eosinophilic asthma may be the result of neutrophilic airway inflammation [2], associated with microbial and/or other irritant exposures. In support, some studies of NEA have reported increased sputum neutrophils, levels of neutrophil-associated mediators, and bacterial endotoxin [5–8]. However, other studies in adults and children have shown little evidence of neutrophilic inflammation in NEA [3, 4]. Neutrophilic asthma (NA) may therefore only be important in a proportion of NEA, and possibly only in some populations.

Initial studies suggested that inflammatory asthma phenotypes are stable [5, 9], although recent reports suggest that temporal changes may be common [10–12]. ICS treatment in particular may affect inflammatory phenotype through a reduction in sputum eosinophils and an increase in neutrophils [10]. Moreover, neutrophils may play a role in poorly controlled asthma, as observed in adults undergoing exacerbation [13]. Nonetheless, effects of asthma control and ICS treatment on inflammatory phenotypes are often not taken into account with most studies assessing airway inflammation only once, and mostly during periods of stable asthma. In addition, many studies have been conducted in hospital settings, in which asthma is likely to be treated with high ICS doses [9]. This may have led to an underestimation of the prevalence of EA in previous studies. Also, assessment of airway inflammation has often not involved methods such as flow cytometry [14], which allow improved definition of inflammatory mechanisms and assessment of the functional status of airway leukocytes, such as neutrophils. Whilst a previous study has suggested that airway neutrophil numbers increase with ICS treatment [10], the effects on airway neutrophil oxidative burst and phagocytosis (which may be associated with effective clearance of debris and dying cells in the airways) [15] has not previously been examined.

In this community based study, we assessed the prevalence and characteristics of inflammatory asthma phenotypes in a general adult population sample. In a preliminary study conducted in a subset of asthmatics, we also examined phenotype stability, and changes in clinical and inflammatory parameters including neutrophil numbers and function (using flow cytometry) following changes in treatment.

## Methods

### Study population

**Fig. 1:**
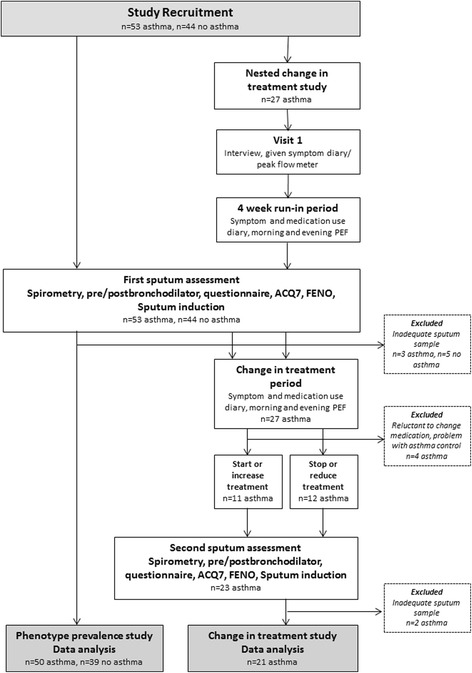
Study plan flow chart. Recruitment of participants, and the assessments involved in the phenotype prevalence and change in treatment components of the study

To obtain a reasonably representative sample of asthmatics we recruited 53 asthmatics and 44 non-asthmatics, aged 17–65, by newspaper advertisement and posters in general practice clinics. As is common for population-based asthma studies, asthmatics were defined on the basis of having both been diagnosed with asthma by a physician and having had wheeze in the last 12 months (with no additional criteria regarding bronchial hyperreactivity, asthma control or lung function results) [14, 16–18]. Non-asthmatics were defined as having had no previous or current asthma diagnosis, no history of wheeze or nocturnal cough in the last 12 months, and forced expiratory volume in 1 s (FEV_1_) > 75% predicted. As respiratory infection is associated with airway neutrophilia, participants who had symptoms or evidence of either upper or lower respiratory infection within four weeks prior to assessment were asked to return at a later date. All participants underwent a respiratory health questionnaire and hypertonic saline sputum induction test. Asthma control status was based on asthma control questionnaire (ACQ) 7 score [19], and as the majority of asthmatics were using inhaled corticosteroids at the time of assessment, was used in preference to asthma severity (as advocated by the Global Initiative for Asthma) [20]. Eighty-nine subjects (91.8%) successfully produced sputum and were included in data analyses examining phenotype prevalence and characteristics (referred to in the remainder of this paper as the “prevalence study”). In a preliminary study of a subset of asthmatics we examined the effects of treatment alterations on phenotype stability, inflammation and neutrophil function, referred to as the “CIT (change in treatment) study” (Figure 1). The CIT study included “only” 27 asthmatics as many were uncomfortable with changing their ICS use/asthma control due to the risk of exacerbations. The study was approved by the Upper South A and Lower South Regional Ethics Committee, New Zealand. All subjects gave written informed consent. The CIT study has retrospective registration at the Australia and New Zealand Clinical Trials Registry (ANZCTR), number 12617001356358.

### Change in treatment study

Twenty-seven randomly selected asthmatics (who stated at initial assessment that they were amenable to asthma treatment change) were included in the CIT study. Participants were interviewed and asked to record morning and evening peak expiratory flow (PEF) using a Vitalograph asmaPLAN (Bucks, UK) peak flow meter, and symptoms and medication use over a 4 week ‘run-in’ period, after which they returned for assessment. Asthma control status was based on ACQ-7 score, PEF and symptom diary. Four subjects left the study as they were subsequently reluctant to change asthma treatment, or had difficulty maintaining asthma control. A change in treatment was made on an individual basis for the remaining 23 subjects by a clinician according to the National Asthma Education and Prevention Program (NAEPP) Expert Panel Report 3 (EPR 3) guidelines [21]. Subjects with adequately controlled asthma had their treatment sub-optimised to a reduced dose of ICS (*n* = 6) or to no ICS (*n* = 6). Subjects with inadequately controlled asthma had their treatment optimised, and either commenced ICS (*n* = 5) or increased ICS dose (*n* = 6). All participants used short-acting beta-agonists (SABA) as required. Changes in treatment (including long-acting beta-agonists (LABA)) for CIT participants are shown in Additional file 1: Table S1.

Subjects returned for a second assessment with a repeat of all previous tests after 4–6 weeks, similar to previous studies assessing the effects of change in treatment on asthma inflammatory phenotypes [10, 11] (Figure 1). Those undergoing treatment sub-optimisation followed a defined safety protocol (see Additional file 1) and asthma treatment was readjusted according to asthma control prior to discharge from the study. Two subjects undergoing treatment sub-optimisation were excluded from CIT study analyses as they were unable to provide an adequate sputum sample at the second assessment.

### Clinical assessment

Skin prick testing (to determine atopy status) was conducted as described in Additional file 1. Spirometric measurements were conducted for all participants according to American Thoracic Society (ATS) criteria [22] using an EasyOne spirometer (NDD Medizintechnik AG, Zurich, Switzerland). NHANES III equations were used for lung function parameters. Exhaled nitric oxide (FENO) measurements were conducted at 50mls/s according to ATS/ERS guidelines [23] using a Hypair FENO analyser (Medisoft, Sorinnes, Belgium).

### Sputum induction

Sputum induction using hypertonic saline and sample processing was conducted as described previously [6] (Additional file 1). Supernatants and cell suspensions were used for subsequent tests, including differential cell counts by light microscopy. Asthmatics with ≥2% eosinophils were classified as EA; those who also had ≥61% neutrophils were classified as mixed granulocytic asthma (MGA). Asthmatics with <2% eosinophils were classified as NEA; those who also had ≥61% neutrophils were classified neutrophilic asthma (NA) and those with <61% neutrophils as paucigranulocytic asthma (PGA) [5].

Measurement of sputum supernatant IL-8 and MMP-9 levels was conducted using sandwich ELISA, and bacterial endotoxin was measured using the Limulus amebocyte assay, as described previously [7]. Neutrophil elastase (NE) was measured as described previously [7] but was undetectable in >90% of samples, and therefore reported as undetected/detected. Endotoxin and NE were only measured in the CIT study.

### Flow cytometry

For the CIT study, remaining sputum cells were used for flow cytometry according to the protocol described in Additional file 1. For neutrophil respiratory burst measurement (paired samples available for seven asthmatics), data were expressed as the percentage of dihydrorhodamine (DHR) 123 (Molecular Probes, Eugene, OR) positive neutrophils after phorbol 12-myristate 13-acetate (PMA, Sigma-Aldrich) stimulation minus negative control. For phagocytosis measurement (paired samples available for 16 asthmatics) data were presented as the percentage of opsonized Texas Red-labelled zymosan A microbeads (Molecular Probes) positive neutrophils after incubation at 37 °C minus percentage bead positive neutrophils in a background control sample. All cell aliquots were also washed and antibody labelled to identify neutrophils by flow cytometry as described previously [14].

### Statistical analysis

Data analyses were performed using STATA version 11.0 (STATA Corp, College Station, TX, USA) and Prism 5 (Graphpad Software Inc., La Jolla, CA, USA). Data are described as median and interquartile ranges (IQR) unless otherwise stated. Univariate analyses were conducted using either student’s t-test or Pearson’s correlation (parametric data), or Mann-Whitney u-testing or Spearman’s correlation (non-parametric data). For the prevalence study, asthmatics were grouped according to either the EA/PGA/NA/MGA or EA/NEA classifications described above to allow comparison with previous studies [2, 5, 10, 11]. For the CIT study, data were initially analysed separately for asthmatics undergoing an increase in treatment (*n* = 11) and asthmatics undergoing a decrease in treatment (*n* = 10). Subsequently, data were combined to increase power and allow comparison of the two visits for all CIT participants when considered as sub-optimised/optimised (*n* = 21). As it has been suggested that EA and NEA have distinct pathologies and response to ICS [3, 4], and that neutrophils may be particularly important in NEA [2], additional analyses were conducted to assess differences associated with CIT in asthmatics characterised as EA/NEA (EA defined as having 2% or greater sputum eosinophils at either assessment). Data were analysed using the paired t-test or Wilcoxon’s matched pairs test as appropriate. Fisher’s exact test was used for categorical data, and Kruskal-Wallis with Dunn’s post-test analysis was used for multiple group comparison.

## Results

Compared with non-asthmatics, asthmatics had higher sputum eosinophil relative cell counts (*p* < 0.0001) and FENO levels (*p* = 0.01), were more likely to be atopic (*p* < 0.0001), had greater bronchodilator reversibility (BDR; *p* = 0.0007), and lower FEV_1_-% predicted and FEV_1_/FVC (both *p* < 0.005) (Table 1). Forty percent of asthmatics were classified as EA and 60% NEA, of which 8% were NA and 52% were PGA; none were classified as MGA. EA was associated with reduced lung function, increased FENO and BDR, and poorer ACQ7 scores; (all *p* < 0.05) compared to PGA (Table 1) or all NEA (data not shown). Eosinophilia correlated with FENO (*r* = 0.74; *p* < 0.0001). With the exception of increased eosinophils in the EA group and increased neutrophils in the NA group (due to phenotype classification), few differences in inflammatory parameters between phenotypes were observed (Table 1). In asthmatics, airway neutrophils were correlated with IL-8 and MMP-9 (*p* = 0.02–0.002; data not shown), but no associations between neutrophils and clinical parameters such as FEV_1_-% predicted, FEV_1_/FVC, BDR or ACQ-7 score (all *p* > 0.1) were observed.

**Table 1:** Clinical characteristics and sputum sample inflammatory characteristics of all participants at baseline

	Neutrophilic asthma (NA)	Paucigranulocytic asthma (PGA)	Eosinophilic asthma (EA)	All asthma	No asthma
Number (% of asthma)	4 (8%)	26 (52%)	20 (40%)	50	39
Sex (female)	4 (100%)	17 (68%)	8 (40%)	30 (60%)	27 (69.2%)
Age	60.5 (40.5–64)	35 (26–47.5)	33.5 (27–43)	35.5 (27–51.8)	37 (27–47)
Atopy	3 (75%)	21 (80.8%)	20 (100%)	44 (88%)	16 (41%)^c^
ACQ7	0.86 (0.25–1.89)	0.57 (0.29–1.00)	1.14 (0.86–1.86)^a^	0.86 (0.43–1.57)	–
FEV1% predicted	92.70 (84.45–100.9)	94.30 (85.25–100.4)	87.25 (79.08–93.90)^a^	91.6 (83.5–98.5)	96.6 (90.5–105.5)^c^
FEV1/ FVC	0.72 (0.68–0.79)	0.79 (0.69–0.84)	0.69 (0.64–0.82)^a^	0.71 (0.65, 0.75)	0.83 (0.77–0.86)^c^
BDR	3.32 (−0.92–13.88)	4 (0–6.00)	13.97 (5.99–19.33)^a^	5.9 (2.06–13.75)	3.0 (1.0–5.0)^c^
FENO (ppb)	28.35 (18.43–86.5)	26.65 (18.63–48.5)	139.5 (95–195.6)^ab^	49.5 (22.4–134.5)	33 (27.3–38.5)^c^
ICS use (%)	4 (100%)	18 (72%)	16 (80%)	38 (76%)	–
TCC/ml	6.07 (0.8–6.12)	1.29 (0.73–2.39)	1.88 (1.2–2.84)	1.40 (0.92–2.84)	1.42 (0.90–2.05)
Viability %(non-squamous cells)	80.74 (69.44–88.24)	65.15 (58.39–74.33)	68.57 (57–81.3)	69.38 (59.5–76)	74.45 (57.5–83)
Sputum eosinophils %	0.12 (0–1.04)	0.47 (0–1.33)	5.8 (3.52–11.78)^ab^	1.358 (0.24–4.61)	0 (0–0.25)^c^
Total sputum eosinophils × 10^4^ ml	1.48 (0–7.89)	0.65 (0–1.48)	12.31 (4.88–29.20)^a^	1.53 (0.37–8.81)	0 (0–0.32)^c^
Sputum neutrophils %	63.14 (61.46–67.47)^a^	24.24 (13.02–33.14)	26.90 (17.43–40.19)^b^	27 (17.21–42.82)	32.1 (19.27–45.43)
Total sputum neutrophils ×10^4^ ml	390.5 (49.03–419.4)	31.27 (11.18–55.04)	48.75 (20.79–96.24)	33.94 (15.7–81.6)	47.51 (10.90–73.10)
Sputum macrophages %	32.37 (30.46–35.59)^a^	68.88 (56.07–77.08)^b^	62.25 (48.93–67.97)	63.95 (49.76–74.58)	60.49 (45.55–78.44)
Total sputum macrophages × 10^4^ ml	185.2 (28.98–188.5)	90.14 (46.87–146.2)	109.7 (67.18–158.7)	96.80 (53.16–160.6)	80.94 (41.71–125.9)
Sputum lymphocytes%	2.36 (1.16–3.99)	1.47 (0.83–2.32)	2.61 (1.69–4.37)^a^	1.94 (0.98–3.14)	2.12 (1.25–3.45)
Total sputum lymphocytes × 10^4^ ml	5.93 (1.39–18.09)	1.83 (0.72–2.92)	4.70 (2.24–8.96)^a^	2.37 (1.15–6.24)	3.15 (1.28–6.22)
Sputum IL-8 (ng/ml)	2.33 (1.47–3.39)	1.77 (1.01–3.08)	2.36 (1.52–3.39)	2.19 (1.26–3.18)	1.62 (1.66–2.33)
Sputum MMP-9	1595 (1084–2943)	528.9 (185.8–1040)	1063 (374–2126)	693.7 (374–1363)	736.7(334.5–1340)

The first three columns report the characteristics of the asthma phenotypes previously described.[5] No MGA phenotype was detected. NA and PGA combined make up the NEA phenotype described in Results [2]

*BDR* change in FEV1% predicted post bronchodilator. Median (IQR) or number (%)

Comparing EA, NA and PGA:

^a^Significantly different (*p* < 0.05) than PGA

^b^Significantly different (*p* < 0.05) than NA

Comparing asthma and no asthma:

^c^Significantly different (*p* < 0.05) than asthma

Following treatment optimisation in the nested preliminary CIT study, fewer asthmatics were classified as EA i.e. of the 11 subjects, EA prevalence reduced from 9 (82%) at first assessment to 6 (55%) at second assessment (Table 2), but this did not reach statistical significance. Correspondingly, following sub-optimisation we found a doubling of EA cases (from two to four i.e. 20% to 40%), more asthmatics with NA (i.e. a change of 3 (30%) from 0 (0%)), and lower levels of MMP-9 and NE (not IL-8), but none of these differences were statistically significant. Overall, 11/21 (52%) asthmatics changed phenotype during the course of the study. When combining those who were initially identified as undergoing sub-optimal treatment with those whose treatment was sub-optimised as part of the study we found that 13/21 (62%) were categorised as EA; this was lower than what was observed during optimised treatment (either as part of the initial assessment or as a result of optimisation) i.e. 8/21 (38.1%), but again this was not statistically significant (Table 2). Sputum bacterial endotoxin levels significantly decreased in the optimisation group (Table 2).

**Table 2:** Alterations in clinical/inflammatory characteristics and inflammatory phenotypes during changes in asthma treatment (CIT study)

	A) Start or increase ICS (*n* = 11)			B) Stop or decrease ICS (*n* = 10)			C) All (*n* = 21) when assessed during:		
	Visit 1 (suboptimal Rx)	Visit 2 (optimised Rx)	Δ	Visit 1 (optimised Rx)	Visit 2 (suboptimal Rx)	Δ	Suboptimal Rx	Optimised Rx	Δ
Female (%)	5 (45%)	–	–	7 (70%)	–	–	12 (57%)	–	–
Age	36 (33, 43)	–	–	44 (27, 56)	–	–	37.0 (31, 55)	–	–
Atopy	10 (91%)	–	–	10 (100%)	–	–	20 (95%)	–	–
ICS dose (μg, budesonide equiv.)	0 (0, 140)	1000 (800, 1000)	1000**	775 (500, 850)	0 (0, 500)	−775**	0 (0, 375)	800 (500, 1000)	800***
ACQ7	1.71 (1.14, 2.14)	0.71 (0.57, 1.07)	−1.00**	0.57 (0.54, 0.90)	1.22 (0.89, 1.82)	0.65**	1.71 (1.07, 2.07)	0.57 (0.57, 1.00)	−1.14***
FEV1% predicted	71.5 (68.2, 82.6)	79.3 (69.5, 82.4)	7.8	82.1 (76.2, 92.4)	85.5 (73.0, 90.9)	3.4	75.6 (69.6, 86.4)	79.3 (74.1, 85.8)	3.7*
FEV1/FVC	0.65 (0.63, 0.68)	0.71 (0.59, 0.78)	0.06	0.74 (0.65, 0.83)	0.73 (0.63, 0.83)	−0.01	0.67 (0.64, 0.76)	0.71 (0.65, 0.78)	0.04
BDR (post-bronchodilator reversibility)% change	15.4 (9.3, 21.8)	11.1 (8.0, 15.5)	−4.3**	5.0 (1, 8.6)	7.7 (1.8, 11.9)	2.7	11.4 (5.3, 16.2)	8.6 (3.9, 12.5)	−2.8**
FENO (ppb)	124 (87–188)	84 (26.6–110)	−40**	44.0 (21.0, 74.0)	62.0 (34.0, 98.0)	18*	89.0 (47.0, 165.5)	51.0 (23.8, 90.0)	−38**
EA (%)	9 (82%)	6 (55%)	−3 (−27%)	2 (20%)	4 (40%)	2 (20%)	13 (61.9%)	8 (38.1%)	−5 (−23.8%)
NEA (%) of which:	2 (18%)	5 (45%)	3 (27%)	8 (80%)	6 (60%)	−2 (−20%)	8 (38.1%)	13 (61.9%)	5 (23.8%)
NA (%)	1 (9%)	1 (9%)	0 (0%)	0 (0%)	3 (30%)	3 (30%)	4 (19.0%)	1 (4.8%)	−3 (−14.3%)
PGA (%)	1 (9%)	4 (36%)	3 (27%)	8 (80%)	3 (30%)	−5 (−50%)	4 (19.0%)	12 (57.1%)	8 (38.1%)
Sputum eosinophils %	6.9 (2.2, 17.2)	3.1 (0.9, 9.1)	−5.4	1.4 (0.6, 1.7)	0.9 (0, 7.4)	−0.5	4.4 (0.7, 15.3)	1.5 (0.8, 4.8)	−2.9
Sputum neutrophils %	27.4 (9.3, 43.7)	30.4 (17.4, 38.7)	3	38.0 (14.5, 45.1)	43.9 (18.9, 63.9)	5.9	35.8 (14, 53.9)	34.5 (17.3, 43.2)	−1.3
TCC/ml	2.33 (0.94, 3.08)	2.08 (1.80, 3.25)	−0.25	1.23 (0.95, 2.07)	0.89 (0.70, 1.47)	−0.34	1.26 (0.74, 2.44)	1.8 (1.17, 3.07)	−0.54
Viability % (non-squamous cells)	69.4 (56.6, 83.1)	78.8 (67.0, 84.4)	9.4	70.9 (63.5, 78.1)	80.2 (65.5, 86.9)	9.3	73.8 (60.3, 85.4)	76.0 (64.0, 81.7)	2.2
Sputum IL-8 (ng/ml)	2.19 (0.58, 2.53)	1.94 (1.19, 4.26)	−0.25	2.53 (1.35, 3.85)	2.05 (1.02, 3.39)	−0.48	2.19 (0.93, 3.11)	2.11 (1.28, 3.57)	−0.08
Sputum MMP-9	411 (259, 2490)	637 (421, 1289)	226	1160 (295, 1584)	1293 (326, 2003)	133	666 (277, 2042)	1094 (376, 1408)	428
NE detected (%)	1 (9%)	0 (0%)	−1 (−9%)	0 (0%)	3 (30%)	3 (30%)	4 (19%)	0 (0%)	−4 (−19%)
Endotoxin (EU/ml)	2027 (1172, 2342)	1235 (546, 1769)	−792*	2227 (720, 4087)	1879 (1477, 3054)	−348	1894 (1354, 2577)	1361 (559, 2650)	−533

ICS dose – average daily dose ICS over recording period in μg budesonide equivalent. Data expressed as number (percentage) or median (IQR). Δ difference between median values of paired groups. ***p < 0.001, ** *p* < 0.01, * *p* < 0.05 when compared with matched group

A and B represent the data derived from the assessments as described (*n* = 11 go from sub-optimised to optimised, n = 10 from optimised to sub-optimised). C represents the data from all 21 CIT participants when data are stratified according to their treatment status

**Fig. 2:**
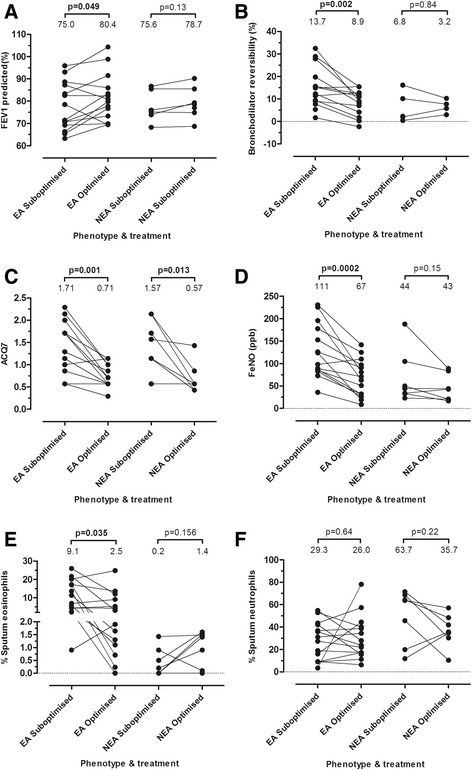
Clinical and sputum sample inflammatory parameters for all CIT participants (*n* = 21). Data represent the assessment in which treatment was considered as sub-optimised or optimised. **a** – FEV1% predicted, **b**- change in FEV1% predicted post-bronchodilator (BDR), **c** – ACQ7, **d**- exhaled NO levels, **e** – change in % sputum eosinophils, **f**-change in % sputum neutrophils. Median data values are expressed at the top of each group

Treatment optimisation was associated with significantly lower BDR (*p* = 0.002), increase in FEV_1_-% predicted (*p* = 0.026), and decrease in FENO (*p* = 0.002) and ACQ7 (*p* = <0.0001; Table 2). When the treatment groups were stratified on the basis of ≥2% eosinophils at either visit as EA/NEA (as it has been suggested that these phenotypes show different responses to ICS) [3, 4], significant lung function and clinical improvements were observed only in the subjects that had been identified as EA on at least one assessment during the CIT study (Fig. 2a-d). The exception to this was a decrease in ACQ7 in NEA with treatment optimisation. Also, there was a significant decrease in eosinophils in the treatment-optimised EA group (Fig. 2e, *p*<0.05) when compared with sub-optimal treatment.

**Fig. 3:**
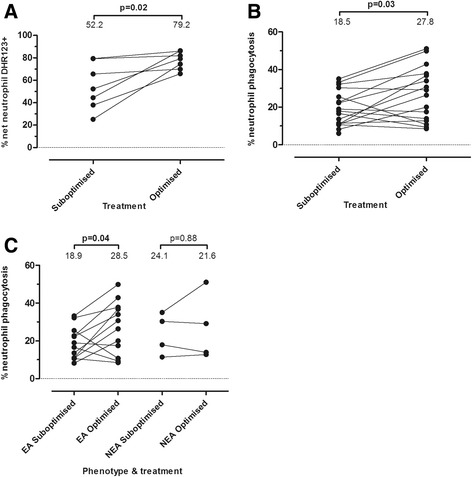
Sputum neutrophil functional parameters (as assessed using flow cytometry). Data represent CIT participants who produced an adequate sample for neutrophil oxidative burst (*n* = 7) or phagocytosis measurement (*n* = 16) at both visits. Groups are stratified on the basis of treatment status (sub-optimised or optimised). **a** – change in % sputum neutrophils undergoing oxidative burst after stimulation with PMA, **b** – change in % sputum neutrophils that are capable of phagocytosing fluorescent zymosan beads in culture at 37 °C, **c**- change in % sputum neutrophils that are capable of phagocytosing fluorescent zymosan beads in culture at 37 °C when stratified into EA/NEA. Median values are expressed at the top of each group

Sputum neutrophils showed a significant increase in oxidative burst after PMA stimulation (*p* < 0.05) and a significant improvement in phagocytosis (*p* < 0.05) (Fig. 3a-b) with optimised treatment. When phagocytosis was compared between EA/NEA groups, a significant increase (*p* < 0.05) in phagocytosis with optimised treatment was only observed in sputum neutrophils from the EA group (Figure 3c). As a loss of neutrophils was observed during in vitro stimulation, we compared this between sub-optimised and optimised treatment, but no significant difference was found (data not shown).

## Discussion

In this community based study of adult asthmatics, in cross-sectional assessment the dominant asthma phenotypes were EA (40%) and PGA (52%). EA was associated with increased airway obstruction, ACQ7 score and BDR. In the nested CIT study, alteration in asthma treatment resulted in more than half of the asthmatics changing phenotype; in particular, sub-optimisation of treatment was associated with a non-significant increased prevalence of EA and NA, whilst optimisation of treatment was associated with an increase in PGA. Treatment optimisation was associated with clinical improvement, reduced eosinophil numbers and increased neutrophil function, statistically significant only in EA.

In our sample of adult asthmatics we found NA in only 8%, consistent with other studies [10, 11]. However, some previous studies have reported that NA is common [5, 13]. We speculate that the higher prevalence of the neutrophilic phenotype found in such studies may be in part due to specific environmental exposures associated with particular occupations or heavy industry [24–26]. Furthermore, NA has previously been reported in association with aging [5, 27]. In agreement, we observed NA only in older individuals, with 3/4 asthmatics classified as NA at any assessment being >60 yrs. of age. Also, consistent with others [28, 29] we found significantly lower FEV_1_, FEV_1_/FEV, and higher ACQ7 scores and BDR in EA.

PGA (which made up the majority of NEA) was associated with significantly higher eosinophil percentages than non-asthmatics despite levels being below the 2% cut-off used to define EA. Therefore, PGA - as defined in this and other studies - may not necessarily be indicative of different asthma pathology, but may represent a less evident form of EA, overlapping phenotypes [30], or be due to treatment effect (discussed further below).

Whilst some studies have shown that NEA is relatively stable over time [5], 52% of asthmatics in our study altered phenotype following changes in treatment. Similar findings have been previously reported. For example, Hancox et al. found that NEA/EA changes occurred in 50 to 100% of adult asthmatics (*n* = 54) in response to changes in ICS therapy [11]. Similar findings have also been described in children [12]. ICS use may therefore contribute to misclassification of NEA, as it may reduce sputum eosinophil numbers below the commonly used cut-point of 2%. Our findings, although not reaching statistical significance, thus support previous suggestions that inflammatory phenotype classification based upon a single assessment may not be valid for all asthmatics [31].

Although some previous reports have shown ICS to be less effective in NEA compared with EA [4, 32, 33], we observed improvements in ACQ7 score in the NEA group with optimised treatment. Other studies have also shown that symptoms improve in NEA with ICS treatment, although EA remains associated with a better response [10, 11]. As noted above, it is possible that some NEA subjects have a degree of ICS-responsiveness, but have undetectable (in sputum) eosinophilic inflammation. Alternatively, ICS may act through suppression of non-TH2 mediated pathways in NEA, such as epithelial cytokine production [34].

ICS use has previously been associated with an increase in sputum neutrophils and neutrophil survival [10], possibly via impairment of apoptosis [35]. We saw no significant changes in airway neutrophil percentages with treatment alterations. However, 33% (3/9) of individuals with PGA changed to NA after a *reduction* in ICS dose. This contrasts with previous findings suggesting that asthma exacerbations after tapered ICS withdrawal are associated with eosinophilic inflammation [36, 37]. However, one previous report suggested that sudden ICS withdrawal, as conducted in our study, may result in neutrophilic exacerbations [38]. It is therefore possible that the nature of airway inflammation post-ICS withdrawal may be dependent upon the kinetics of withdrawal.

We observed that neutrophil function (oxidative burst and phagocytosis) was enhanced with treatment optimisation, with the greatest effect observed in EA. Although earlier asthma studies have investigated sputum phagocyte function [39, 40], we believe that this is the first study assessing neutrophil function in response to changes in asthma treatment. The association between improved neutrophil function and treatment optimisation is intriguing. It is possible that improved neutrophil phagocytosis may be one of the mechanisms by which asthma treatment leads to a reduction in eosinophilic inflammation, possibly through improved efferocytosis [15]. Alternatively, improved neutrophil function may have affected the airway microbiome [41] as suggested by significantly reduced bacterial endotoxin (a strong pro-inflammatory agent) levels (Table 2). It is also possible that impairment of neutrophil apoptosis (described above) may have altered neutrophil maturation status, leading to the altered functional phenotype observed. However, larger longitudinal investigations are required to confirm these findings and more comprehensively determine the effect of medication on neutrophil function; in particular, whether the functional changes observed are directly relevant to the improvement of asthma symptoms, whether treatment leads to alteration of peripheral neutrophil function (blood samples were not available for assessment in the current study) or if treatment is indirectly affecting neutrophil function through modulation of other cell populations, such as macrophages or eosinophils. Also, although not addressed here, there is a possibility that the hypertonic saline challenge or sputum sample processing procedure may directly result in altered or activated neutrophil phenotype. Previous studies have reported increased expression of the activation markers CD11b and CD66 on sputum neutrophils when compared with blood or broncho-alveolar lavage neutrophils [42, 43], but it is not clear if this is due to the airway environment or sampling procedure. However, in the current study, as all samples were processed in the same manner, it is unlikely that the observed differences could be due to either saline challenge or sputum processing.

There were some limitations to this study. Firstly, although asthmatics reported a doctor’s diagnosis of asthma and recent symptoms, as we did not use objective tests (such as bronchodilator reversibility or airway hyperreactivity) to confirm asthma diagnosis, it is possible that some misclassification may have occurred. However, we consider that any bias introduced as result will be minimal given that this approach, which has been used in many previous studies [14, 16–18, 26], generally compares well with more clinical definitions of asthma [16, 17]. Also, in some cases it has been shown to be better than more objective measures such as airway hyperreactivity [18]. Indeed, there are a number of issues with objective testing for confirmation of asthma diagnosis in a community based setting, particularly given the inherently variable nature of the condition, and when the majority of asthmatics are not treatment naïve (76% of asthmatics in the current study were using ICS at the time of assessment). This (amongst other reasons) has led to recent recommendations that asthma be considered on the basis of symptoms rather than pathophysiology [44]. Secondly, as the majority of asthmatics were undergoing ICS therapy, and there were safety concerns about total ICS withdrawal in some cases in the CIT study, it is possible that ICS treatment may be “cloaking” physiological responses, as well as eosinophilic inflammation. Thirdly, the relatively large number of cells required to investigate cell function meant that only samples with larger cell yields could be assessed. For this reason, respiratory burst was assessed in relatively few samples (*n* = 7 for respiratory burst and 16 for phagocytosis assessment). This may have resulted in selection bias. However, our data suggest that there is no significant difference between sputum total cell count between asthmatics and non-asthmatics, and therefore this is unlikely to be a major issue. Fourthly, treatment changes were limited to a 4–6 week period (similar to previous studies [10, 11] and varied somewhat among CIT study participants as asthma management was considered on a case-by-case basis i.e. subjects did not receive a uniform reduction or increase in ICS dose (Additional file 1: Table S1), and in cases in which participants were using combined ICS/LABA inhalers, LABA was also altered. All participants were also using SABA as required. This may have led to some variation in the effect on (particularly eosinophilic) inflammation and/or asthma control, as there are reports that some SABA (in particular terbutaline) may have a permissive effect on airway inflammation, and their use may be associated with an increase in airway eosinophils [45]. However, the evidence for this is mixed, and may not be the case for all SABA [46, 47]. Fifthly, the asthmatics studied were recruited from the general population, and as such had varying levels of asthma control and severity at assessment which makes the interpretation of the results more difficult. Finally, the relatively small number of participants included in the CIT study (due to the reluctance of some study participants to undergo CIT) may have led to non-significant findings in terms of inflammatory phenotype changes. Despite the latter two limitations, the trends observed (i.e. increased EA with suboptimised treatment, reduced eosinophils, improvement in asthma control and increased NEA with optimised treatment) were generally similar to those previously described in studies in which ICS was withdrawn or added to asthma therapy [10, 11].

## Conclusions

In this sample of adult asthmatics, NEA was common and associated with less severe disease, with only a small proportion of NEA demonstrating evidence of neutrophilic inflammation. This study also showed that treatment optimisation was associated with reduced sputum eosinophils, improved symptoms and lung function, and enhanced neutrophil function, but a significant reduction in EA could not be demonstrated.

## Additional file

Additional file 1: Methods S1. Details of the safety protocol for the sub-optimisation of treatment, skin-prick testing, sputum induction and processing, and sputum flow cytometry. Table S1. Number of participants in the change in treatment study using ICS and/or LABA. (DOCX 30 kb)

## Abbreviations

ACQ: Asthma control questionnaire
BDR: Bronchodilator reversibility
CIT: Change in treatment
DHR: Dihydrorhodamine
EA: Eosinophilic asthma
FENO: Fractional exhaled nitric oxide
FEV_1_: Forced expiratory volume in 1 s
FVC: Forced vital capacity
ICS: Inhaled corticosteroid
LABA: Long acting β2- agonist
MGA: Mixed granulocytic asthma
NA: Neutrophilic asthma
NE: Neutrophil elastase
NEA: Non-eosinophilic asthma
PEF: Peak expiratory flow
PGA: Paucigranulocytic asthma
PMA: Phorbol 12-myristate 13-acetate
SABA: Short acting β2- agonist
