# Supplementary material for: Effects of treatment changes on asthma phenotype prevalence and airway neutrophil function
Source: BMC Pulm Med. 2017 Dec 4;17:169. doi: 10.1186/s12890-017-0511-6 (PMC5715501; doi:10.1186/s12890-017-0511-6)
Supplement: Additional file 1: — Methods S1. Details of the safety protocol for the sub-optimisation of treatment, skin-prick testing, sputum induction and processing, and sputum flow cytometry. Table S1. Number of participants in the change in treatment study using ICS and/or LABA. (DOCX 30 kb) [file 12890_2017_511_MOESM1_ESM.docx]

**Additional File 1**

Effects of treatment changes on asthma phenotype prevalence and airway neutrophil function

**Methods**

**Safety protocol for sub-optimisation of treatment**

Subjects undergoing treatment sub-optimisation were given a 3 day prescription of prednisone 40mg/day and instructed to contact the investigators if they had a fall in morning PEF over 7 days of > 10% of baseline, a drop in morning or evening PEF of > 20% on 2 consecutive days, an increase in daily use of reliever of > 3 puffs above baseline, an increase in night-time wakening of > 3 nights or more/week above baseline, or distressing or intolerable asthma symptoms. If none of these occurred, they returned within 4-6 weeks for further assessment. Throughout this period, reliever medication was used as required, and participants monitored their PEF twice daily and recorded their asthma symptoms and reliever use. No participants required prednisone during the study.

**Skin prick testing**

Skin prick testing was conducted using a panel of aeroallergens; house dust mite, tree mix, grass mix, cat and dog dander, Alternaria tenuis and Penicillium mix (Hollister-Stier Laboratories, Spokane, WA, USA). Positive atopy status was determined as the presence of at least one wheal of >3mm. Histamine and saline were used as positive and negative controls respectively

**Sputum induction and processing**

Spirometry was performed before and 15 minutes after 400ug salbutamol via spacer. Subjects then proceeded to hypertonic saline sputum induction. Aerosolised sodium chloride (4.5%w/v) was administered orally using an ultrasonic nebuliser (DeVilbiss Ultraneb 2000) for increasing intervals from 30 seconds to 4 minutes, to a total of 16 minutes. At the end of the session participants were encouraged to produce a sputum sample into a sterile plastic container.

Sputum samples were processed by plug selection and DTT treatment. Total cell count and viability were determined using light microscopy. The sample was then centrifuged (350xg, 8 minutes), supernatant aspirated, and the cell pellet resuspended in RPMI 1640 (Invitrogen, Auckland, New Zealand), 10% foetal calf serum (FCS, Invitrogen), and 1% penicillin-streptomycin at 1x10^6^ cells/ml. A fraction of this cell suspension (75µl) was centrifuged in a cytospin column at 44xG for 5 minutes. The slide produced was air dried, methanol fixed and stained with the Diff-Quik stain set (Dade Behring, Deerfield, IL). Four hundred non-squamous cells were counted by light microscopy. Samples were deemed acceptable if this number of non-squamous cells could be counted and squamous cell contamination was less than 30%.

**Sputum flow cytometry**

For neutrophil respiratory burst measurement, three 100µl (2 x 10^5^) cell aliquots per subject were added to low-adherence 96 well plates with 0.5µM (DHR)123 (Molecular Probes, Eugene, OR). Plates were incubated (37°C, 5 mins) and immediately quenched on ice. One aliquot was kept on ice (negative control) and one aliquot was incubated (37°C, 30 mins) with 1ng phorbol 12-myristate 13-acetate (PMA) (Sigma-Aldrich) and then quenched on ice (activated sample). For phagocytosis assessment (paired samples available for 16 asthmatics), two 100µl (2 x 10^5^) cell aliquots per subject were added to wells of chilled low-adherence 96 well plates alongside 10:1 concentration of opsonized Texas Red-labelled zymosan A microbeads (Molecular Probes). One aliquot was kept on ice (background control) and one aliquot was incubated (37°C, 60 mins) and then quenched on ice (positive sample).

Following incubation with fluorochrome-labelled monoclonal antibodies on ice for 30 mins (to identify neutrophils), samples were washed, incubated with live/dead fixable blue (Molecular Probes) on ice for 30 mins and washed again with FACS buffer. Labelled aliquots were then resuspended in 150µl FACS buffer/150µl neutral buffered formalin (Sigma).

Samples were stored on ice and analysed on a BD LSRII flow cytometer within 3 hours. Appropriately-labelled compensation beads (BD) and unlabelled cells were used for compensation.

Table S1. ICS/LABA use in CIT study

| Optimised treatment | | |
| --- | --- | --- |
| Number of subjects | ICS | LABA |
| 5 | Started | None |
| 3 | Increased dose | None |
| 3* | Increased dose | Increased dose |
| Sub-optimised treatment | | |
| Number of subjects | ICS | LABA |
| 5* | Stopped | Stopped |
| 4* | Reduced dose | Reduced dose |
| 1 | Stopped | Continued |

*Using combined ICS/LABA (e.g. seretide, symbicort)
